# Light Signaling Regulates Aspergillus niger Biofilm Formation by Affecting Melanin and Extracellular Polysaccharide Biosynthesis

**DOI:** 10.1128/mBio.03434-20

**Published:** 2021-02-16

**Authors:** Wenjun Sun, Ying Yu, Jiao Chen, Bin Yu, Tianpeng Chen, Hanjie Ying, Shengmin Zhou, Pingkai Ouyang, Dong Liu, Yong Chen

**Affiliations:** a National Engineering Research Center for Biotechnology, College of Biotechnology and Pharmaceutical Engineering, Nanjing Tech University, Nanjing, China; b State Key Laboratory of Materials-Oriented Chemical Engineering, College of Biotechnology and Pharmaceutical Engineering, Nanjing Tech University, Nanjing, China; c School of Chemical Engineering and Energy, Zhengzhou University, Zhengzhou, China; d State Key Laboratory of Bioreactor Engineering, School of Biotechnology, East China University of Science and Technology, Shanghai, China; The Hebrew University of Jerusalem

**Keywords:** light signaling, *Aspergillus niger*, biofilm, melanin, MAPK signaling pathway

## Abstract

Light is an important signal source in nature, which regulates the physiological cycle, morphogenetic pathways, and secondary metabolites of fungi. As an external pressure on Aspergillus niger, light signaling transmits stress signals into the cell via the mitogen-activated protein kinase (MAPK) signaling pathway. Studying the effect of light on the biofilm of A. niger will provide a theoretical basis for light in the cultivation of filamentous fungi and industrial applications. Here, the characterization of A. niger biofilm under different light intensities confirmed the effects of light signaling. Our results indicated that A. niger intensely accumulated protective mycelial melanin under light illumination. We also discovered that the *RlmA* transcription factor in the MAPK signaling pathway is activated by light signaling to promote the synthesis of melanin, chitin, and other exopolysaccharides. However, the importance of melanin to A. niger biofilm is rarely reported; therefore, we knocked out key genes of the melanin biosynthetic pathway—*Abr1* and *Ayg1*. Changes in hydrophobicity and electrostatic forces resulted in the decrease of biofilm caused by the decrease of melanin in mutants.

## INTRODUCTION

The filamentous fungal biofilm is a structured microbial community composed of mycelia wrapped in an extracellular matrix (ECM) that adheres to each other. In industrial fermentation, researchers have discovered that fungal biofilms are more resistant to environmental stress and shorten the fermentation cycle (1). However, to date, little research has been conducted on the biofilm of Aspergillus niger in industrial processes. This study aimed to establish a connection between light signaling and A. niger biofilm.

Filamentous fungi differ from bacteria and yeast in biofilm formation due to the special structure and function of their spores and mycelium (2–4). *Aspergillus* biofilm formation includes spore colonization, germination, hyphal elongation, reproductive development, ECM production, and maturation and diffusion of biofilms (5, 6). In general, the main factors affecting biofilm formation of A. niger are the spore attachment and hyphae adhesion (7). In spore morphology, hydrophobic and electrostatic interactions represent critical factors affecting spore attachment. In mycelium morphology (8), extracellular polysaccharides, such as galactosaminogalactan (GAG) and α-1,3-glucan, mediate adhesion and are major factors affecting biofilm (9, 10). The mature biofilm of *Aspergillus* is rich in ECM, composed of extracellular polysaccharides, hydrophobins, melanin, and environmental DNA (eDNA), to protect its own mycelium from adverse external factors, such as heat shock, oxidative stress, and nutritional deficiencies (10, 11).

There are numerous factors that affect biofilms, such as environmental factors, signaling pathway regulation, and quorum sensing molecules (12). Light is a ubiquitous signal in nature, which may vary concerning intensity and wavelength in different places. Therefore, a few wavelength-specific photoreceptors have evolved in fungi (13). Although fungi cannot directly harness light as an energy source (14), fungi can convert light energy into chemical signals in cells, thereby enabling each organism to adapt to its habitat (15). Visible light can also be used as an indicator of co-occurring environmental stress, thereby improving stress resistance and survival. This coincides with the concept of biofilm. However, the mechanism of light action is complex. Furthermore, light signaling has attracted more attention in fungi, especially in the production and development of secondary metabolites. However, many details regarding the effect of light on metabolic processes have yet to be elucidated. In particular, the relationship between light signaling and biofilms is a worthy research subject.

In eukaryotes, the mitogen-activated protein kinase (MAPK) signaling pathway is an important component involved in diverse cellular processes, which are essential for signal transmission, integration, and amplification (16). Fungi sense and respond to various signals through the ubiquitous and evolutionarily conserved MAPK signaling pathway. As a source of fungal stress, light signaling is also an external factor that activates the MAPK signaling pathway. Yu et al. discovered that *Aspergillus* sp. uses the *SakA*(*Hog1*) MAPK pathway to perceive light signaling (17). In recent years, the relationship between the MAPK signaling pathway and biofilm has also been reported in Aspergillus fumigatus. The loss of MAP kinases *MpkA*, *MpkC*, and *SakA* affected the cell surface and ECM in biofilm, while mutants reduced adhesion to polystyrene and fibronectin-coated plates in A. fumigatus (18, 19). It was also reported that *MpkA* and *Hog1* functioned upstream of *RlmA* and *RlmA* and acted as a transcription factor for the corresponding cell wall pressure, the expression of chitin and glucan, and the melanin biosynthetic pathway (20–23). 1,8-Dihydroxynaphthalene (DHN)-melanin produced by A. niger induces a black appearance, and the biosynthetic pathway of A. niger DHN-melanin involves *Alb1*, *Ayg1*, *Arp2*, *Arp1*, *Abr1*, and *Abr2* (24, 25).

A light-sensing system in filamentous fungi was represented by Aspergillus nidulans and Neurospora crassa. We observed certain phenotypic differences between A. niger grown under light or dark environments. A. niger intensely accumulated protective mycelial melanin under light illumination. In this study, it was observed for the first time that light signaling could regulate the MAPK signaling pathway in A. niger to promote melanin production. Melanin has also been shown to play a role in biofilm of A. niger. Overall, light signaling may mediate the formation of biofilm in A. niger. Therefore, using light signaling to regulate biofilm formation in A. niger may provide an effective strategy to increase the yield of target products. These findings provide a theoretical basis for light signal regulation of biofilm in filamentous fungi.

## RESULTS

### Light signaling affected A. niger biofilm formation.

**FIG 1:**
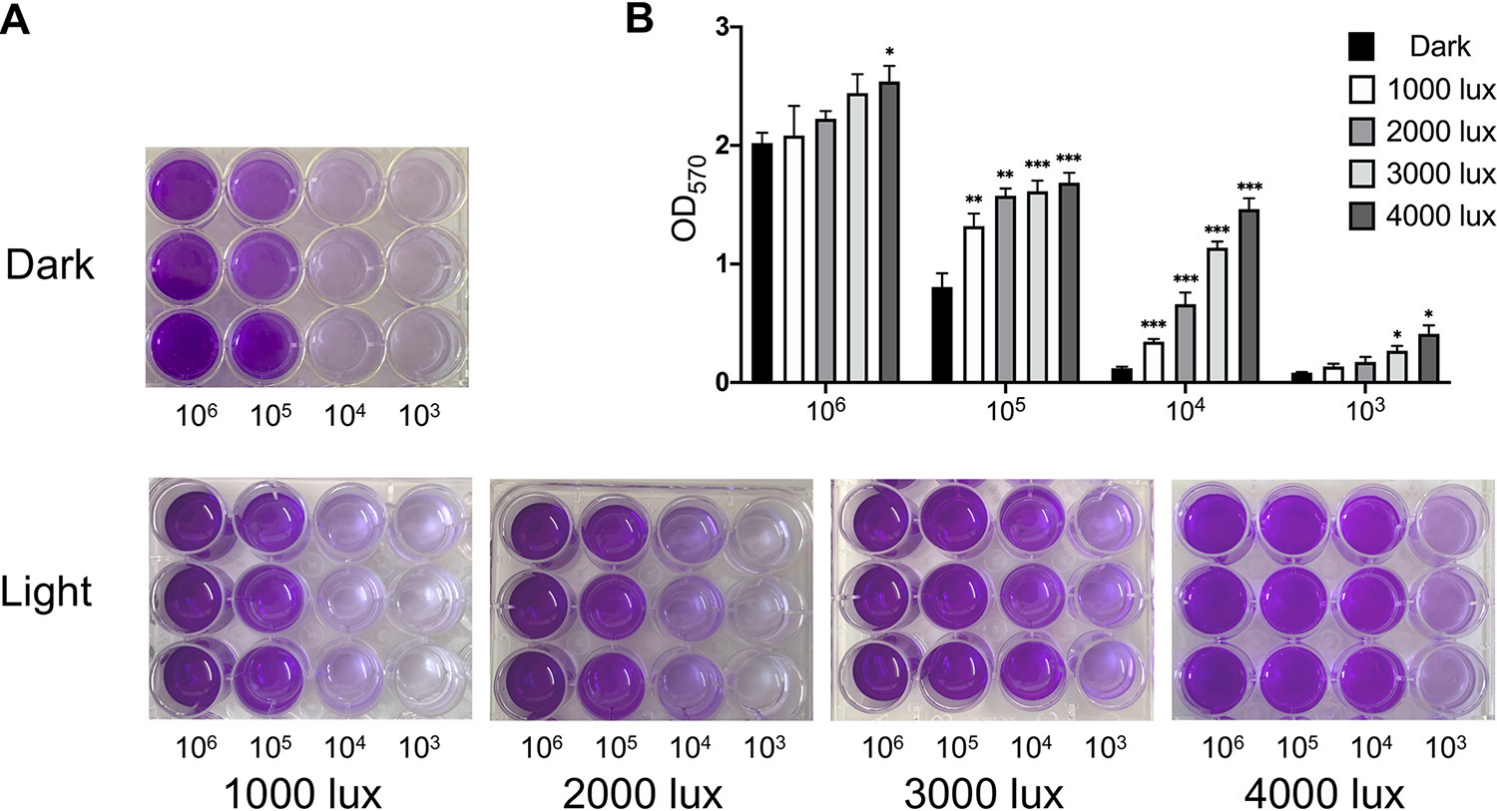
Effect of light on biofilm formation in A. niger. (A) Various amounts of A. niger wild-type spores were inoculated into a 24-well plate, incubated at 30°C in the dark, exposed to light intensity of 1,000 to 4,000 lx for 36 h, and then photographed after CV staining. (B) The corresponding OD_570_ value in the 24-well plate. The values represent the means and standard deviations of three independent experiments. ***, *P < *0.001; **, *P < *0.01; *, *P < *0.05; two-way ANOVA.

**FIG 2:**
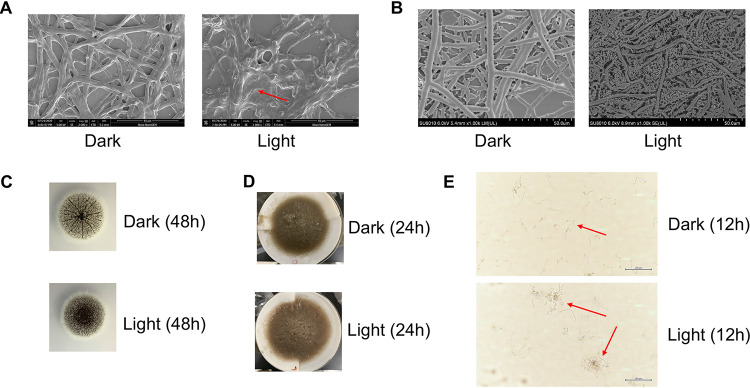
Appearance and microscopic morphology of A. niger grown in light or darkness. (A) Biofilm on round coverslip after light or dark culture; image was taken by Nova NanoSEM. Scale bar, 40 μm. (B) Biofilm on round coverslip after light or dark culture; image taken by cryo-SEM. Scale bar, 50 μm. (C) A. niger colony growing on PDA solid medium under light or dark conditions. (D) Different colors of A. niger mycelium collected under light or dark conditions (YPD liquid medium). (E) Microscope graphics of A. niger hyphae in YPD liquid medium under light or dark conditions. Scale bar, 200 μm.

Visible light exerts a stressful effect on nonphotosynthetic organisms (26). Therefore, it is of interest to study the relationship between light and biofilm in fungi. To evaluate the ability of A. niger to form biofilms on a solid surface, biofilms grown in 24-well plates inoculated with different concentrations of A. niger spores were quantified using crystal violet (CV) assays. The results showed that light with an increasing intensity from 1,000 lx to 4,000 lx could increasingly promote biofilm formation (Fig. 1A). There was a significant difference in optical density at 570 nm (OD_570_) values when the light intensity measured above 2,000 lx. The difference was particularly obvious when the inoculum amount measured 10^5^ and 10^4^ spores/well. The biofilm showed a 9.4-fold and 13.5-fold increase under 3,000 lx and 4,000 lx (Fig. 1B), respectively, when the inoculum amount measured 10^4^. The morphology of A. niger under light or dark conditions was analyzed using a scanning electron microscope (SEM) to further verify this phenomenon. The result in Fig. 2A shows that the adhesion degree of the mycelium under light conditions is higher. In contrast, hyphae lines under dark conditions were clearer. Cryo-SEM was used to observe the samples more realistically to avoid the drying progress of conventional SEM. The ECM between the mycelium under light conditions was more abundant (Fig. 2B), which obviously differs from that under dark conditions. This finding also provides a foundation for the subsequent study of light signaling effects on biofilms.

In order to exclude the effect of light signaling on the growth of the mycelium, we conducted a normal growth experiment. As Fig. 2C shows, A. niger observed on the solid plate medium shows the same colony diameter size under light or dark conditions, but the phenotype showed darker and denser spores under light conditions. There is no significant difference in the dry weight of the mycelium collected at the end of the shake flask culture (results not shown), but there is a significant difference in the color of the mycelium (Fig. 2D). Thus, our evidence indicates that light exerts a minimal effect on the growth of A. niger. We also found that the aggregation degree of hyphae in the shake flask culture for 12 h increased under light conditions, as observed with a microscope (Fig. 2E).

**FIG 3:**
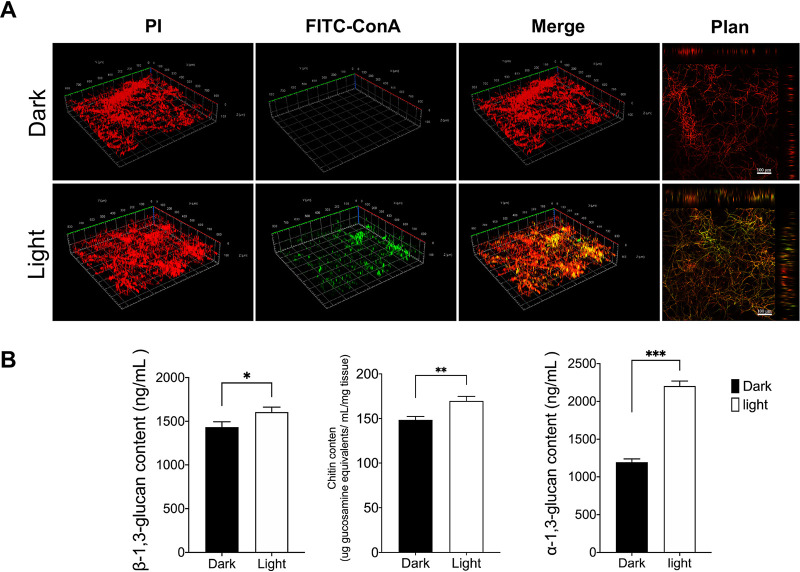
Impact of light sensing on ECM. (A) Immunofluorescence staining images of A. niger biofilm mycelium and polysaccharides taken with a confocal laser-scanning microscope under light or dark conditions. Red indicates PI-stained DNA, and green indicates FITC-ConA-stained exopolysaccharides. Scale bar, 800 μm by 800 μm and 100 μm. (B) The content of β-1,3-glucan, chitin, and α-1,3-glucan. The values represent the means and standard deviations of three independent experiments. ***, *P < *0.001; **, *P < *0.01; *, *P < *0.05; using the Student’s *t* test.

**FIG 4:**
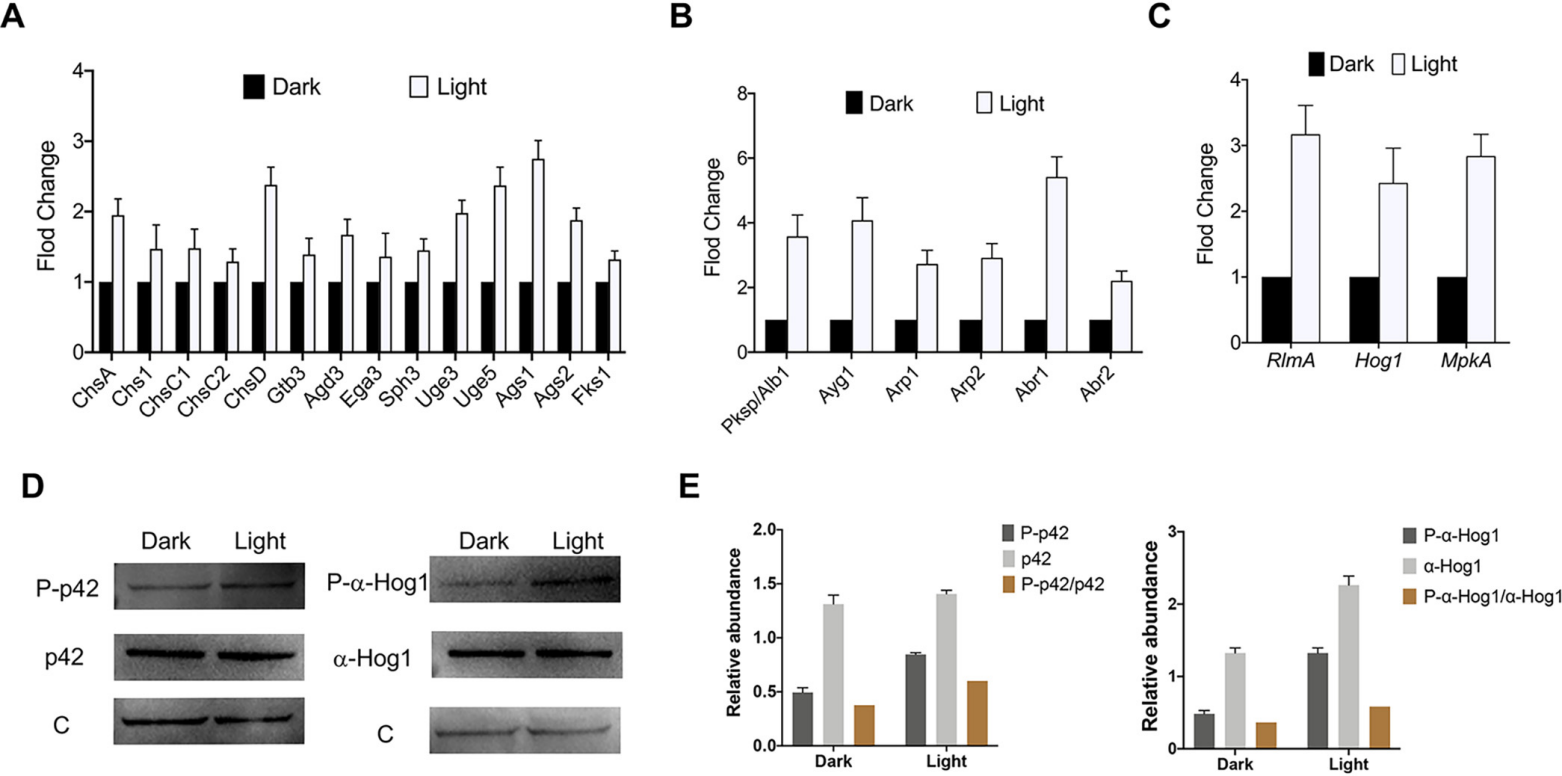
Elevated expression of related genes and proteins under light. (A) Expression of genes involved in the synthesis of chitin, GAG, GM, α-1,3-glucan, and β-1,3-glucan. (B) Expression of genes in melanin biosynthesis. (C) Expression of key genes in the MAPK signaling pathway. (D) Western blot results of *Hog1* and *MpkA* protein expression and phosphorylation level under light and dark conditions. (E) Relative abundance of *MpkAp* and *Hog1p* and phosphorylated MAPK ratio under light or dark conditions. P-p42/p42 and P-α-Hog1/α-Hog1 represented the ratio of phosphorylated proteins to total proteins, calculated by means. The values represent the means and standard deviations of three independent experiments. ***, *P < *0.001; **, *P < *0.01; *, *P < *0.05; using the Student’s *t* test.

The ECM was composed of galactomannan (GM), galactosaminogalactan (GAG), α-1,3-glucans, melanin, antigens, and hydrophobins (27). Polysaccharides are the main components. Therefore, we performed immunofluorescence assays on A. niger biofilm mycelium and extracellular polysaccharides and captured three-dimensional (3D) images using a confocal laser scanning microscope. Figure 3A shows the difference in polysaccharide content. Chitin, α-1,3-glucan, and β-1,3-glucan contents were investigated, and results showed that the content of these polysaccharides increased to a certain extent under light conditions; in particular, α-1,3-glucan increased by 45.9% (Fig. 3B). Subsequently, genes involved in GAG and GM synthesis (*Gtb3*, *Agd3*, *Ega3*, *Sph3*, *Uge3*, and *Uge5*), glucan biosynthesis (*Fks1*, *Ags1*, and *Ags2*), and chitin synthesis (*ChsA*, *ChsB*, *ChsC1*, *ChsC2*, and *ChsD*) were quantitatively analyzed using reverse transcription-quantitative PCR (qRT-PCR). Figure 4A shows the increased expression of these genes under light conditions.

### Light signaling affected A. niger melanin biosynthesis pathways by affecting the MAPK signaling pathway.

**FIG 5:**
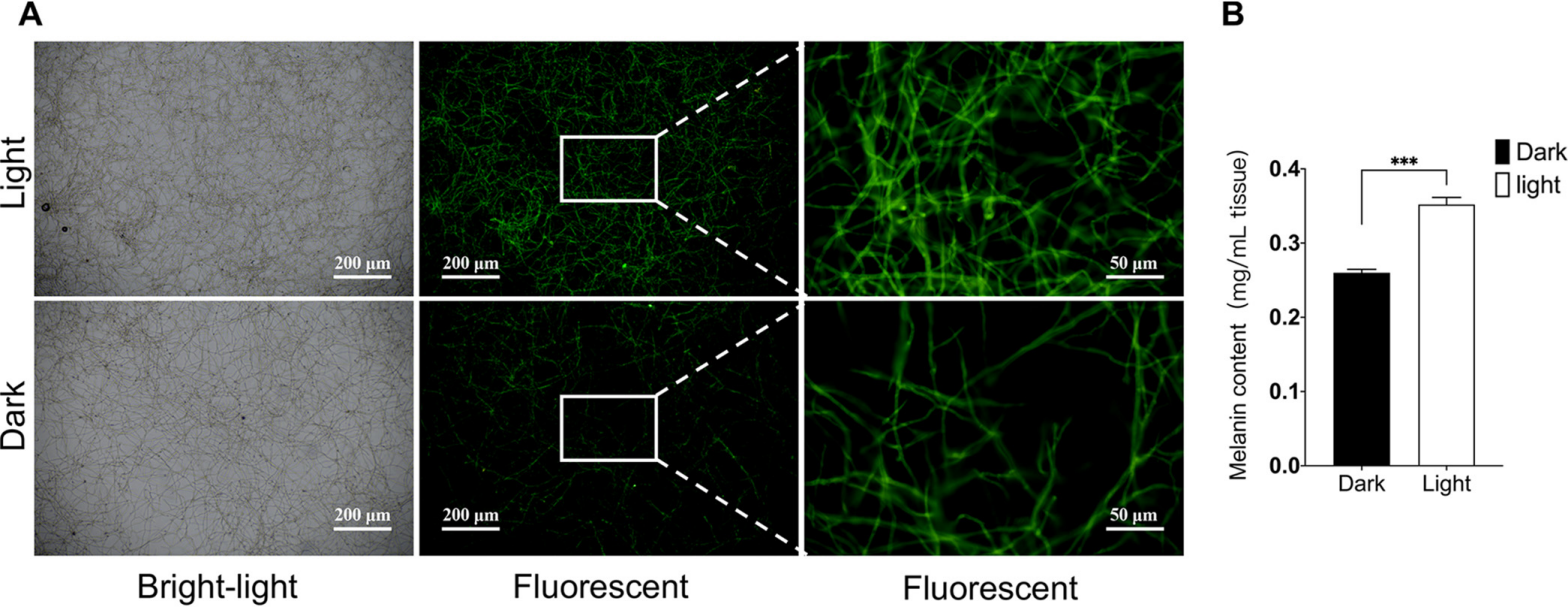
Light promotes the production of melanin. (A) Microscope images of A. niger under light or dark conditions. Scale bar, 200 μm and 50 μm. (B) The content of melanin. The values represent the means and standard deviations of three independent experiments. ***, *P < *0.001; **, *P < *0.01; *, *P < *0.05; using the Student’s *t* test.

It was reported that light signaling could regulate the MAPK signaling pathway and affect melanin formation (23, 28). The expression levels of key genes in the MAPK signaling pathway and melanin biosynthetic pathway were investigated when A. niger was grown under light or dark conditions. Expression levels of *Hog1*, *MpkA*, and *RlmA* increased by 2.4-fold, 2.8-fold, and 3.2-fold, respectively, under light conditions (Fig. 4C). The expression of *Abr1* and *Ayg1* increased significantly, increasing by 4.1-fold and 5.4-fold under light, respectively (Fig. 4B). Through melanin content detection and immunofluorescence experiments, we observed a noteworthy difference in melanin levels when the amount of mycelium remained constant (Fig. 5A). We also observed a 26.1% increase in melanin under light conditions (Fig. 5B). Therefore, *Abr1* and *Ayg1* were knocked out to verify the fundamental role of melanin in biofilms.

In order to further prove that light signaling affects the high osmolarity glycerol (HOG)-MAPK and cell wall integrity (CWI)-MAPK pathways, Western blotting was used to detect the protein expression of *Hog1* and *MpkA* and corresponding phosphorylation level. The key kinases involved in HOG-MAPK and CWI-MAPK pathways appeared increased under light conditions, and light signaling induced the phosphorylation of HOG-MAPK and CWI-MAPK (Fig. 4D). In addition, quantitative determination of *Hog1p* and *MpkAp* and the corresponding phosphorylation level also displayed a positive effect of light signaling on A. niger MAPK pathways (Fig. 4E).

### *A. niger* Δ*Abr1* and Δ*Ayg1* strains increased hydrophobicity and reduced zeta potential.

**FIG 6:**
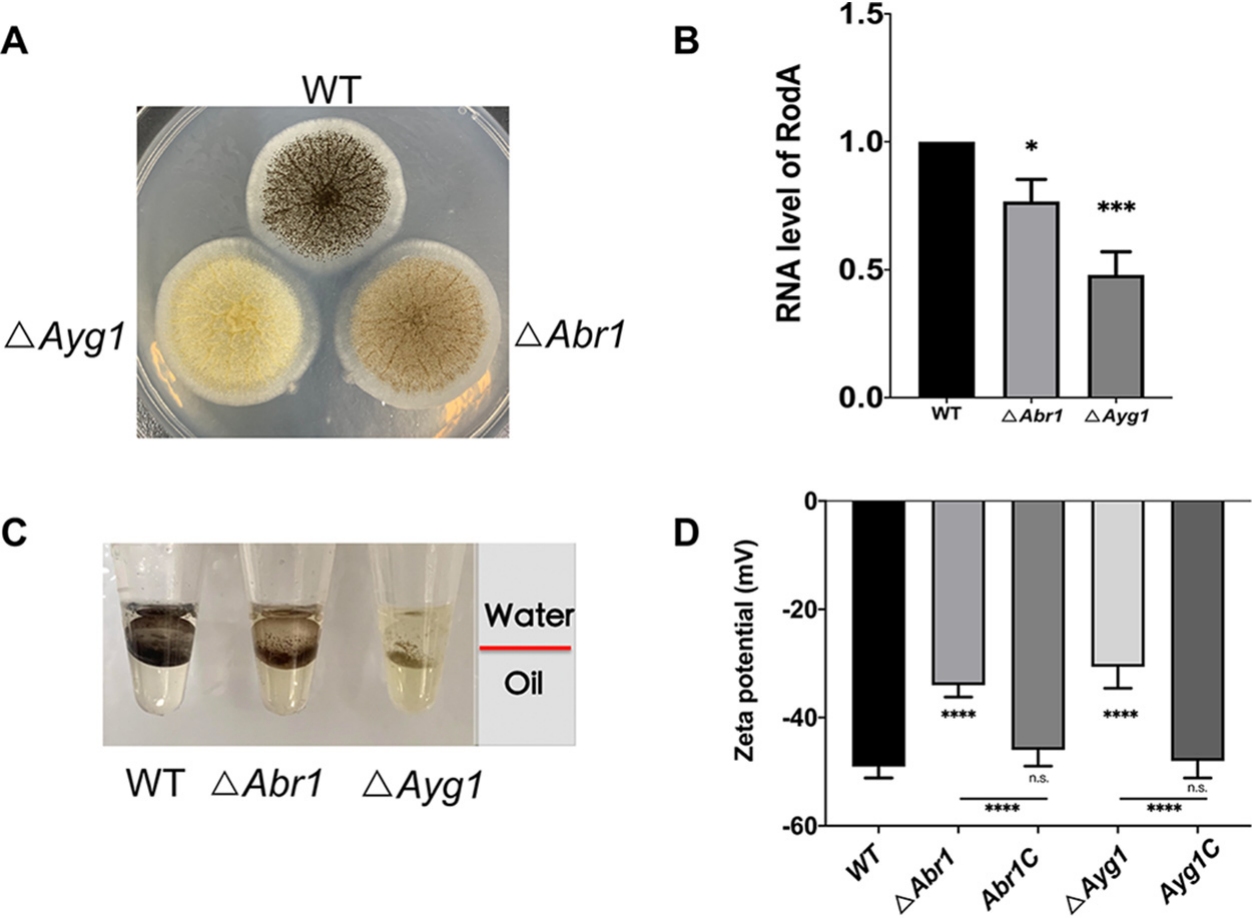
Characterization of hydrophobicity and electrostatic force of A. niger Δ*Abr1* and Δ*Ayg1* strains. (A) Growth of Δ*Abr1* and Δ*Ayg1* strains on normal plates. (B) RNA level of *RodA* in Δ*Abr1* and Δ*Ayg1* strains. (C) Zeta potential of Δ*Abr1* and Δ*Ayg1* spores. (D) Hydrophobicity detection of Δ*Abr1* and Δ*Ayg1* spores. The values represent the means and standard deviations of three independent experiments. ***, *P < *0.001; **, *P < *0.01; *, *P < *0.05; using the Student’s *t* test.

After observing the same amount of Δ*Abr1* and Δ*Ayg1* spores on a solid plate, the color change of the spores can be clearly observed after 48 h (Fig. 6A). The aggregation of spores is driven by electrostatic and hydrophobic interactions (29), which derive from the carboxyl group of the melanin layer and hydrophobin on the spore wall, respectively. Spore aggregation determines the initial stage of biofilm formation (7). Hydrophobicity experiments indicate that Δ*Abr1* and Δ*Ayg1* are less hydrophobic than wild type (WT) (Fig. 6C), and the *RodA* expression level was also consistent with the results in Fig. 6B. The zeta potential test showed that Δ*Abr1* and Δ*Ayg1* exhibited reduced negative charges compared with WT (Fig. 6D).

### *ΔAbr1* and *ΔAyg1* affected biofilm formation and cell wall integrity.

**FIG 7:**
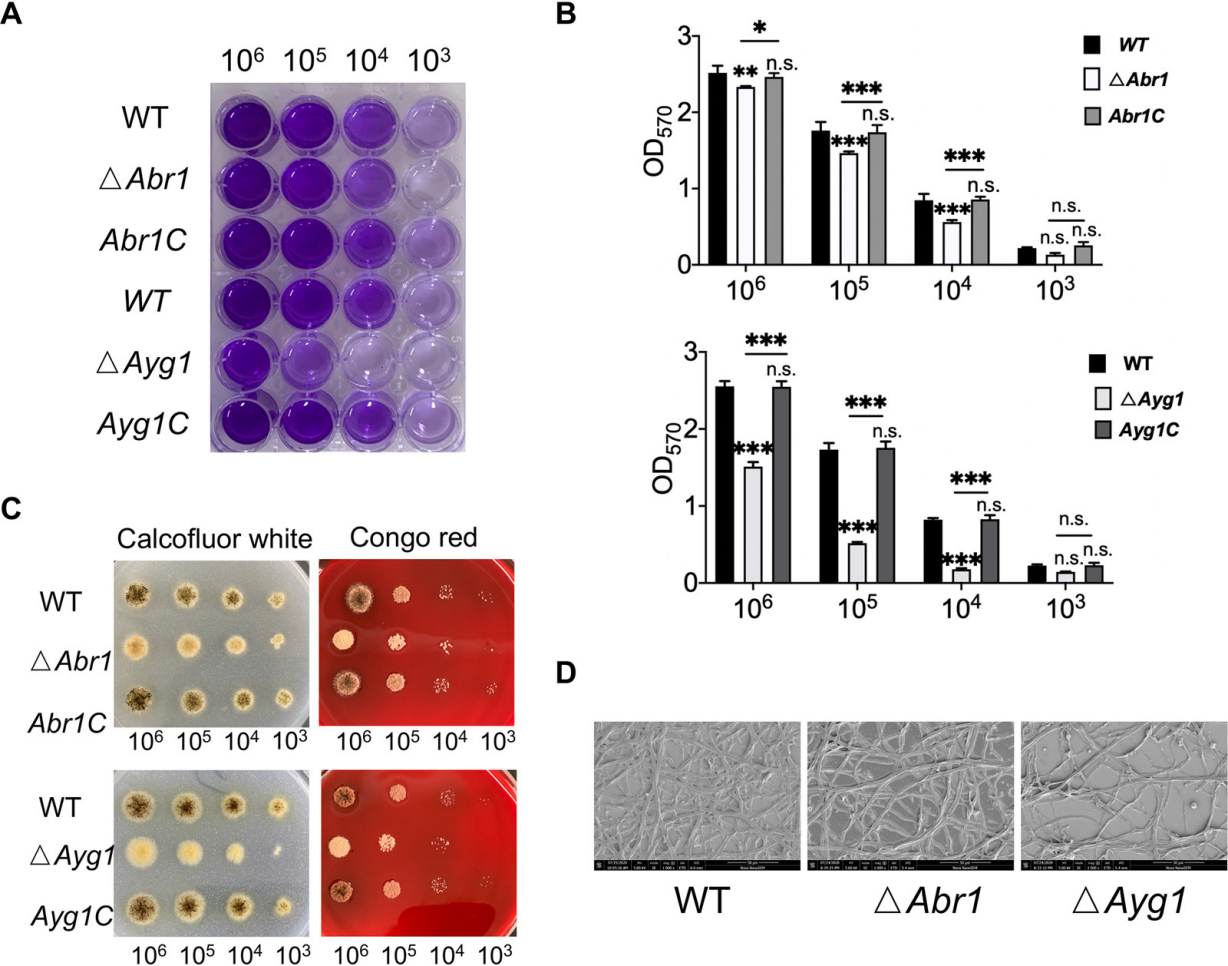
Biofilm formation ability and resistance to cell wall disrupters in A. niger Δ*Abr1* and Δ*Ayg1* strains. (A and B) Image and OD_570_ of A. niger Δ*Abr1* and Δ*Ayg1* strain CV assay results. (C) resistance of A. niger Δ*Abr1* and Δ*Ayg1* strains to Congo red and Calcofluor white. (D) SEM images of A. niger Δ*Abr1* and Δ*Ayg1* strains. Scale bar, 50 μm. The values represent the means and standard deviations of three independent experiments. ***, *P < *0.001; **, *P < *0.01; *, *P < *0.05; using the Student’s *t* test.

We conducted CV assays to analyze the ability of mutants to form biofilms. The results showed that the biofilm-forming ability of Δ*Abr1* and Δ*Ayg1* became weak and that *Abr1*C and *Ayg1*C restored the ability of biofilm formation to a certain extent (Fig. 7A and B). It has been reported that melanin plays a role in maintaining the integrity of the cell wall (25, 30). Congo red and Calcofluor white can be widely used as indicators of cell wall defects. The sensitivity of mutants and WT to these two cell wall indicators were detected. The results clearly showed that Δ*Abr1* and Δ*Ayg1* are more sensitive to Congo red and Calcofluor white (Fig. 7C). This result indicated that the lack of melanin alters the integrity of the cell wall. We observed lower levels of mycelium in Δ*Abr1* and Δ*Ayg1* than in WT, using SEM (Fig. 7D). These results suggested that the degree of melanin reduction was positively correlated with the degree of biofilm reduction.

## DISCUSSION

Biofilms play important roles in the environment and medical and industrial fields (31, 32), which promotes interest in biofilms as a research topic. However, research on biofilm mechanisms in the industrial field is relatively scarce. Here, we researched the effects of light on *Aspergillus* biofilm. Light is an important signal molecule in the environment, which regulates the physiological cycle, morphological changes, and fungal metabolites (15, 33). Among bacteria, it has been reported that Acinetobacter baumannii could form biofilms under dark conditions, while the presence of blue light will inhibit the formation of biofilms (34). In fungi, there were also relevant reports showing that red or blue light affected the development of biofilms and the production of extracellular polysaccharides in Candida albicans (34, 35). Although extensive studies have reported that fungi can sense light signaling, there were no direct reports on white light signaling and biofilms in filamentous fungi. Therefore, an attempt was made to analyze how light signals affect A. niger biofilm.

Several experiments were conducted to investigate the role of light signaling to determine whether it affected the biofilm of A. niger. First, a CV assay was performed. The effect of light signaling on the formation of A. niger biofilm was confirmed using this classic semiquantitative experiment. Second, ECM formation was observed under light conditions using cryo-SEM. Results showed that light signaling promotes the production of A. niger ECM and promotes A. niger biofilm formation.

In *Aspergillus* sp., the effect of GAG and α-1,3-glucan on biofilm has been widely reported (36, 37). It is clearly pointed out that these polysaccharides are similar to cell wall components. α-1,3-Glucan plays a vital role in mycelial aggregation in biofilm, and GAG mediates many virulence-related characteristics, including adhesion to host cells and other substrates and the formation of biofilms. Immunofluorescence experiment results indicate that extracellular polysaccharide production increased under light conditions. α-1,3-Glucan content increased by 45.9%, and β-1,3-glucan and chitin appeared slightly increased. In addition, the expression of extracellular polysaccharide-related genes in biofilms increased by at least 1.3-fold and up to 2.75-fold. Simply put, light signaling promoted the production of polysaccharides in the ECM of A. niger biofilm.

Spores grown on the plate and the mycelium grown on the shake flask exhibited different colors under light or dark conditions. We estimated that melanin may play an important protective and component role in A. niger biofilm. Therefore, melanin content was determined, and genes playing key roles in the process of melanin biosynthesis were quantitatively analyzed. It was found that light can indeed promote the expression of genes related to the melanin biosynthesis pathway, resulting in elevated production of melanin. Therefore, we concluded that light signaling promotes melanin production.

This study mainly proves the positive effect of light signaling on biofilms, while also focusing on melanin. It has been reported that melanin is essential for the correct assembly of the conidial cell wall of A. fumigatus (25). Moreover, melanin not only was a virulence factor but also affected the normal functioning of other virulence factors, such as adhesins and hydrophobin (38). Reports regarding the relationship between melanin biosynthesis and biofilm formation in *Aspergillus* sp. are lacking. Here, we studied the effects of *Abr1* and *Ayg1* in the melanin biosynthesis pathway on biofilm. The results showed that the charge and hydrophobicity of Δ*Abr1* and Δ*Ayg1* were reduced, correlating with the decreasing biofilm trend. Since melanin is an important component of the A. niger cell wall (25), we analyzed the susceptibility of mutants to Congo red and Calcofluor white and found that Δ*Abr1* and Δ*Ayg1* are more sensitive to these two cell wall disrupters and that Δ*Ayg1*, which produces less melanin, displayed higher sensitivity. The results of CV and SEM assays show that Δ*Ayg1* contains less biofilm than Δ*Abr1*. Therefore, our results indicate that a lack of melanin will severely affect cell wall integrity and biofilm formation.

**FIG 8:**
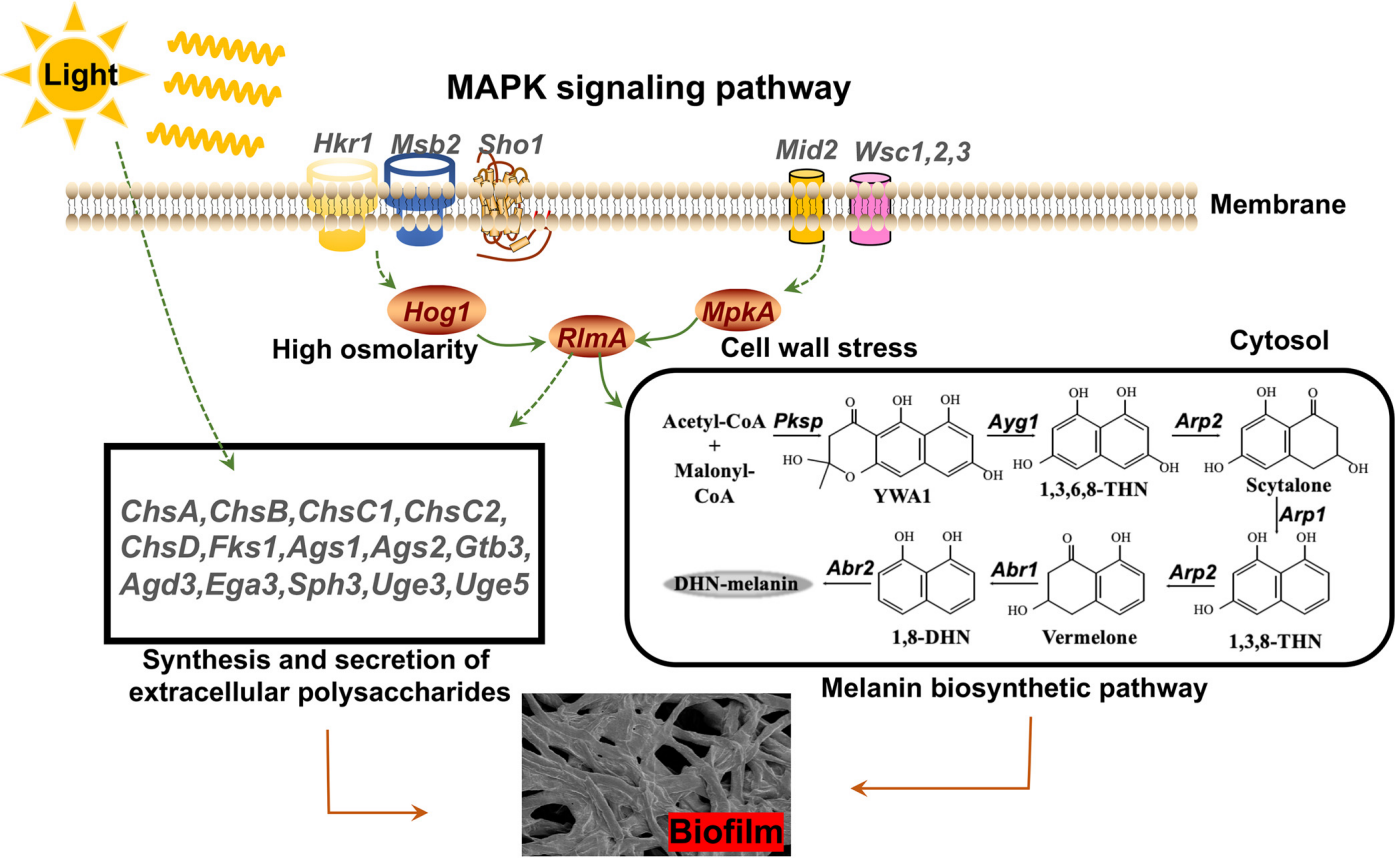
Schematic diagram of light signaling-mediated MAPK signaling pathway involved in A. niger biofilm formation. Solid arrow, activation; dotted arrow, indirect effect.

Amplifying the external pressure signal through the MAPK signaling pathway may also enable light sensing in A. niger because light signaling represents an external pressure on fungi. In order to prove that light signaling governs MAPK signaling pathways, qRT-PCR and Western blotting were carried out. We found that the expression levels of *Hog1* and *MpkA* were increased under light conditions by 2.4-fold and 2.8-fold, respectively. Next, the phosphorylation status of *Hog1* and *MpkA* were investigated, and the results demonstrated that both protein expression and corresponding phosphorylation levels were increased. Furthermore, the proportion of phosphorylated *MpkAp* and *Hog1p* under light is significantly increased. Therefore, we proved that light signaling could activate HOG-MAPK and CWI-MAPK signaling pathways in A. niger. It was reported that the lack of *RlmA* leads to reduced biofilm formation in A. fumigatus (39). As a transcription factor downstream of the HOG-MAPK and CWI-MAPK signaling pathways, *RlmA* could mediate the synthesis of chitin and glucan, as well as the melanin biosynthesis pathway (40, 41). It was also reported that *RlmA* could induce the expression of *Ags1* in A. niger (22). To test the hypothesis that light promotes ECM production by increasing *RlmA* expression, we used qRT-PCR and observed that the *RlmA* transcript increased 3.2-fold. Overall, this study confirmed that light signaling could activate the HOG-MAPK and CWI-MAPK signaling pathways and, secondly, could activate the expression of *RlmA* transcription factors to promote the production of melanin and polysaccharides in A. niger. Therefore, we have drawn a schematic diagram indicating the light signaling-mediated MAPK signaling pathway that regulates A. niger biofilm (Fig. 8).

In conclusion, we found that 3,000 lx light intensity has little effect on the normal growth of A. niger, but it will cause visible differences in the color of spores and hyphae. A series of biofilm characterization experiments confirmed that light could promote the formation of ECM of biofilms, and protective mycelial melanin played a role in resisting light stress. This was the first time that the connection between light signaling and biofilm has been established in A. niger. To our knowledge, the promotion of biofilm formation in fungi by light signaling is also a novel finding. Nevertheless, it is a pity that this discovery has not been applied to fermentation, but it will also be the goal of subsequent research. In the long-term evolution process, the fungus has formed an extremely fine and complete photoreceptive system, which can sense the presence or absence of light, the direction and quality of light, the intensity of light, and the length of the photoperiod, in order to better adapt to the environment. Therefore, it will be interesting to observe the effects of other factors, such as light wavelength, light intensity, light direction, and light period, on the formation of A. niger biofilm.

## MATERIALS AND METHODS

### Strains and media.

In the present study, A. niger SJ1 (WT) was preserved in China Center for Type Culture Collection (Wuhan, China) under the deposit number CCTCC M201911. A. niger SJ1 was cultivated on defined minimal medium (MM) (42), yeast extract-peptone-dextrose (YPD) medium, or peptone-dextrose agar (PDA) medium. For the knockout gene, 60 μg/ml of hygromycin B (31282; Sangon Biotech, China) was added to PDA medium as a *hph* selection marker, and 10 mM acetamide and 15 mM cesium chloride were used in the MM instead of NaNO_3_ for the *amdS* selection marker for the complement gene. *Abr1* and *Ayg1* knockout fragments were constructed using the *hph* gene as a selection marker. A complementing plasmid was constructed using A. nidulans *amdS* as a selection marker. The original plasmid was pAN-*amdS*, constructed by replacing the *hph* gene in pAN7-1 with the *amdS* gene, which was preserved in our laboratory. The *gpdA* gene was used as a promoter and the *trpC* gene was used as a terminator to construct a complementary plasmid expressing *Abr1* and *Ayg1*, respectively. Gene knockout fragments and complementary plasmids were transformed into mutant strains by polyethylene glycol (PEG)-mediated protoplast transformation. The mutant strains used in subsequent experiments were stably passaged more than five times and were verified by colony PCR and qRT-PCR analysis. A schematic diagram of DNA fragments and plasmid construction is shown in Fig. S1 and S2 in the supplemental material. The PCR primers and verification results are indicated in Tables S1 to S3 and Fig. S3 in the supplemental material.

10.1128/mBio.03434-20.1 FIG S1 Schematic diagram of homologous recombination gene knockout. Download FIG S1, PDF file, 0.2 MB. Copyright © 2021 Sun et al. 2021 Sun et al. https://creativecommons.org/licenses/by/4.0/ This content is distributed under the terms of the Creative Commons Attribution 4.0 International license.

10.1128/mBio.03434-20.2 FIG S2 Schematic diagram of plasmid construction. Download FIG S2, PDF file, 0.1 MB. Copyright © 2021 Sun et al. 2021 Sun et al. https://creativecommons.org/licenses/by/4.0/ This content is distributed under the terms of the Creative Commons Attribution 4.0 International license.

10.1128/mBio.03434-20.3 FIG S3 qRT-PCR verification of the mutant strains and complemented strains. The values are the means and standard deviations of three independent experiments. Download FIG S3, PDF file, 0.3 MB. Copyright © 2021 Sun et al. 2021 Sun et al. https://creativecommons.org/licenses/by/4.0/ This content is distributed under the terms of the Creative Commons Attribution 4.0 International license.

10.1128/mBio.03434-20.4 TABLE S1 Sequence of the oligonucleotide primers used for gene knockout in this study. Download Table S1, PDF file, 0.1 MB. Copyright © 2021 Sun et al. 2021 Sun et al. https://creativecommons.org/licenses/by/4.0/ This content is distributed under the terms of the Creative Commons Attribution 4.0 International license.

### Biofilm formation on plastics and normal growth on medium.

A. niger biofilm formation was evaluated using the classic CV assay, performed as previously described with minor modifications (43). Fresh A. niger conidia (10^6^, 10^5^, 10^4^, and 10^3^) were inoculated on a 24-well microtiter plate (Corning, USA) with 1-ml MM and cultured for 36 h at 30°C under 1,000-lx, 2,000-lx, 3,000-lx, and 4,000-lx light intensity, with dark as a contrast. Subsequently, the plate was washed three times with phosphate-buffered saline (PBS) to remove free mycelium. Thereafter, the biofilm was stained with 1 ml 0.1% crystal violet solution at room temperature for 10 minutes, and wells were repeatedly washed with PBS and air dried. Acetic acid (1 ml; 100%) was added to each well, and wells were gently shaken at room temperature for 30 minutes to elute crystal violet. Finally, a microplate reader (SpectraMax Paradigm) was used to read the absorbance at 470 nm. The CV assay was also used to analyze differences in biofilm-forming ability between mutants (Δ*Abr1*, Δ*Ayg1*, *Abr1*C, and *Ayg1*C) and WT A. niger. Next, the OD_470_ was determined, as mentioned above. The light intensity used in subsequent light experiments was 3,000 lx.

WT A. niger spores (10^6^) were one-point inoculated on PDA solid medium, cultured at 30°C for 24 h under light or dark conditions, and then observed. WT A. niger spores (10^7^) were inoculated into a 500-ml conical flask containing 50 ml YPD liquid medium and cultured at 30°C and 220 rpm under light or dark conditions. Sampling was conducted after 12 h to observe the growth difference using an optical microscope. After 24 h, the mycelia were removed and washed thrice with PBS, and differences were observed. WT and Δ*Abr1* and Δ*Ayg1* spores (10^6^) were spot-planted on PDA solid medium, cultured at 30°C for 48 h, and observed.

### Determination of melanin, α-1,3-glucan, β-1,3-glucan, and chitin content in mycelium.

Samples cultured in 24-well plates for 36 h under light or dark conditions were removed and washed thrice with PBS to remove free hyphae. Biofilm hyphae were collected using physical methods and ground into a powder after liquid nitrogen quick freezing and vacuum freeze drying. Specimens were homogenized thoroughly using a homogenizer after adding 1-g hyphae powder to 9-g PBS (pH 7.2 to 7.4). Samples were centrifuged for 20 minutes (3,000 × *g*/min), and supernatants were carefully collected. The Microorganism Melanin ELISA kit (Jiangsu Baolai Biotechnology, Nanjing, China) was used to determine melanin content, the microorganism α-glucans ELISA kit was used to determine α-1,3-glucan content, and the microorganism β-flucans ELISA kit was used to determine β-1,3-glucan content in the biofilm (44–46). Chitin was measured as previously described (43).

### SEM analysis and cryo-SEM analysis.

WT A. niger spores (10^6^) were inoculated into 24-well plates containing 8-mm round coverslips and 1-ml MM and then cultured for 36 h at 30°C under light or dark conditions. Subsequently, the round coverslip was washed three times with PBS to remove free mycelium and then removed to obtain samples. Sample morphologies were observed using the FEI Nova nano-scanning electron microscopy 450 (Nova NanoSEM, Prague, Czech Republic) after liquid nitrogen quick freezing, vacuum freeze drying, and gold spraying.

In order to reduce the influence of drying and dehydration on the observation of samples, we also adopted cryo-SEM to observe the samples. Samples were obtained as mentioned above. Processed samples were loaded into the scanning electron microscope (SU8010) refrigerated transport system (pp3010T). After being precooled with liquid nitrogen for 5 minutes, the sample was transferred to the freezing sample preparation chamber, vacuumed, and sublimated at −70°C. After gold spraying, the sample was transferred to a scanning electron microscope for observation and photographing (47).

In order to compare the difference between the mutants (Δ*Abr1* and Δ*Ayg1*) and WT A. niger, we also used SEM to observe the samples, using the method mentioned above.

### Protein extraction and Western blotting.

As previously described, fresh conidia were incubated in 50-ml YPD medium at 30°C in a rotatory shaker (220 rpm) for 24 h under light or dark conditions. The mycelia collected by filtration were washed thrice with PBS and then quick frozen in liquid nitrogen. The lysis solution was added for protein extraction. The mycelial powder was obtained using a homogenizer and collected into centrifuge tubes. Protein extraction buffer (0.8 ml) containing protease inhibitors and 1 mM phenylmethylsulfonyl fluoride (PMSF) were added into each centrifuge tube and incubated on ice for 20 min. The supernatant was collected via centrifugation, and the protein content was measured using the bicinchoninic acid (BCA) assay. Subsequently, protein samples of the same concentration were loaded onto a 12% (wt/vol) SDS polyacrylamide gel and transferred to a nitrocellulose membrane. The phosphorylation of *Hog1* and *MpkA* was examined using anti-phospho-p38 MAP kinase antibodies and anti-phospho-p42 antibodies (Cell Signaling Technology, USA; dilution, 1:1000). Anti-*Hog1p* C-terminal antibody (Santa Cruz Biotechnology, USA; dilution 1:500) and anti-p42 antibodies were used against *Hog1* and *MpkA*, respectively. A β-Actin antibody was used as a control, and anti-rabbit IgG (whole molecular) peroxidase antibody (Sigma-Aldrich) was used to detect all primary antibodies, except anti-*Hog1p* C-terminal antibody. Goat anti-mouse IgG (H&L) horseradish peroxidase (HRP) was used to detect the anti-*Hog1p* C-terminal antibody (17, 48). The experiment was repeated three times.

### Immunofluorescence.

Samples were fixed with 1.5% glutaraldehyde for 1.5 h and then stained with propidium iodide (PI; Sigma; excitation wavelength, 535 nm; emission wavelength, 615 nm) and fluorescein isothiocyanate-labeled concanavalin A (FITC-ConA; Sigma; excitation wavelength, 495 nm; emission wavelength, 515 nm) in the dark. PI embedded in double-stranded DNA fluoresces red, while FITC-ConA binds to glucose and mannose residues of cell wall polysaccharides and fluoresces green. Confocal laser-scanning microscopy graphics were captured using a Leica TCS SP5 II instrument.

SP-D, a C-type lectin, binds to melanin pigment on the surface of *Aspergillus* sp. (49, 50). Glutaraldehyde-fixed A. niger biofilm samples were incubated with surfactant protein D (SP-D) (1 h; 37°C). Next, samples were washed twice and incubated overnight with primary anti-human SP-D antibody (5 μg/ml; R&D Systems) at 4°C, then washed and incubated with secondary anti-mouse IgG-FITC (dilution 1:200; Sigma) on ice for 1 h, and finally observed using a fluorescence microscope.

### Hydrophobicity assay and zeta potential determination.

The hydrophobicity of A. niger spores was tested according to the method described previously. Glyceryl tributyrate (50 μl) was added to a 50-μl suspension containing 10^8^ spores in a 1.5-ml centrifuge tube and then observed after incubation at room temperature for 24 h. The color depth in the hydrophobic layer indicates the hydrophobicity of the spores (43, 51).

The spore concentration of WT, Δ*Abr1*, Δ*Ayg1*, *Abr1*C, and *Ayg1*C was diluted to 10^6^/ml (pH 6.0), and zeta potentials of WT, Δ*Abr1*, Δ*Ayg1*, *Abr1*C, and *Ayg1*C were determined using a ZetaPlus instrument from Brookhaven (7).

### Cell wall stress resistance test.

Congo red and Calcofluor white are widely used as indicators to show cell wall defects (52). WT spores of different concentrations (10^6^, 10^5^, 10^4^, and 10^3^), Δ*Abr1*, Δ*Ayg1*, *Abr1*C, and *Ayg1*C were inoculated on MM medium containing Congo red (1000 μg/ml) or Calcofluor white (400 μg/ml) for 48 h at 30°C.

### qRT-PCR analysis.

The mycelium inoculated on a 24-well plate under light or dark conditions was washed with PBS thrice to remove floating hyphae, and mycelium was then collected using physical methods. Next, RNA was extracted from mycelia using the TaKaRa MiniBEST universal RNA extraction kit, according to the manufacturer’s instructions. cDNA was obtained via reverse transcription of RNA according to the HiScript III RT SuperMix for qPCR (+gDNA wiper) (Vazyme, China) instructions. The qRT-PCR was performed on a StepOnePlus real-time PCR system (Applied Biosystems, USA) using 2× ChamQ universal SYBR qPCR master mix (Vazyme, China). The reaction and calculations were performed according to standard protocols. Each sample had three parallel and negative controls. The expression level of the gene was calculated according to the comparative threshold cycle (2^−ΔΔ^*^CT^*) method. Actin was chosen as the internal reference gene (53), Primer 5 software was used to select primers, and primers used for analysis are listed in Table S3.qRT-PCR experiments were performed, as indicated above, to verify the difference in hydrophobicity between WT and Δ*Abr*1, Δ*Ayg1*, *Abr1*C, and *Ayg1*C and to verify the expression of the hydrophobin *RodA*.

10.1128/mBio.03434-20.5 TABLE S2 Sequence of the oligonucleotide primers used for plasmid construction in this study. Download Table S2, PDF file, 0.1 MB. Copyright © 2021 Sun et al. 2021 Sun et al. https://creativecommons.org/licenses/by/4.0/ This content is distributed under the terms of the Creative Commons Attribution 4.0 International license.

10.1128/mBio.03434-20.6 TABLE S3 Genes and primers used for qRT-PCR. Download Table S3, PDF file, 0.1 MB. Copyright © 2021 Sun et al. 2021 Sun et al. https://creativecommons.org/licenses/by/4.0/ This content is distributed under the terms of the Creative Commons Attribution 4.0 International license.

### Statistical analysis.

All experiments were done at least in triplicate. Statistical significance was analyzed by one- or two-way analysis of variance (ANOVA) or paired *t* test with Prism software (GraphPad software, San Diego, CA). The values are the means and standard deviations of three independent experiments and *P* values generated by the Student’s *t* test (n.s., not significant at *P* > 0.05; ***, *P < *0.001; **, *P < *0.01; *, *P < *0.05).
