# Supplementary figures and images for: Light Signaling Regulates Aspergillus niger Biofilm Formation by Affecting Melanin and Extracellular Polysaccharide Biosynthesis
Source: mBio. 2021 Feb 16;12(1):e03434-20. doi: 10.1128/mBio.03434-20 (PMC8545115; doi:10.1128/mBio.03434-20)

**Figure S1.** Schematic diagram of homologous recombination gene knockout.

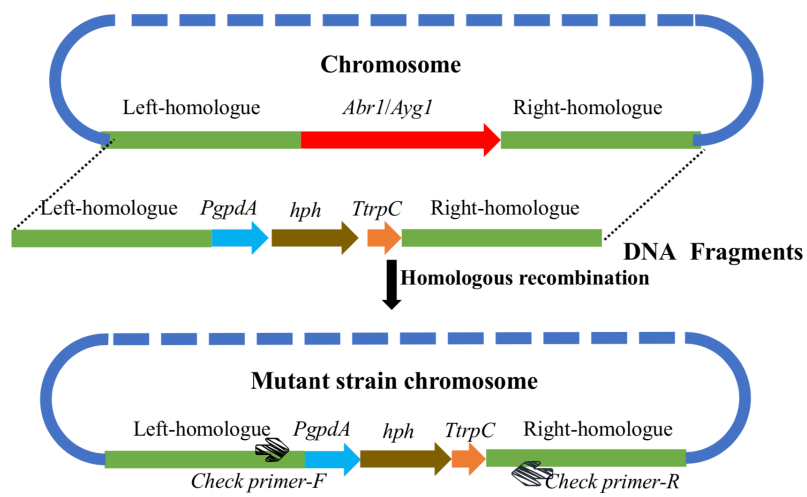

Supplement: FIG S1 [file mbio.03434-20-sf001.pdf]

**Figure S2.** Schematic diagram of plasmid construction.

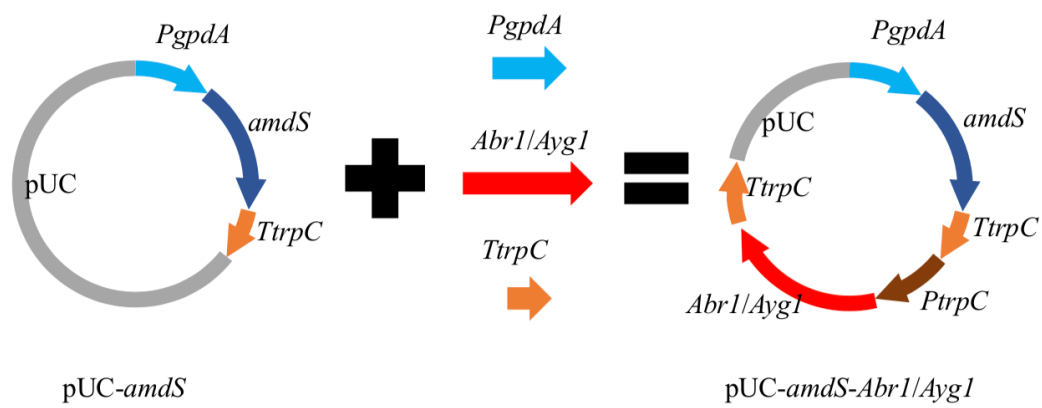

Supplement: FIG S2 [file mbio.03434-20-sf002.pdf]
