# Supplementary material for: Light Signaling Regulates Aspergillus niger Biofilm Formation by Affecting Melanin and Extracellular Polysaccharide Biosynthesis
Source: mBio. 2021 Feb 16;12(1):e03434-20. doi: 10.1128/mBio.03434-20 (PMC8545115; doi:10.1128/mBio.03434-20)

Figure S3. qRT-PCR verification of the mutant strains and complemented strains. The values are the means and standard deviations of three independent experiments.

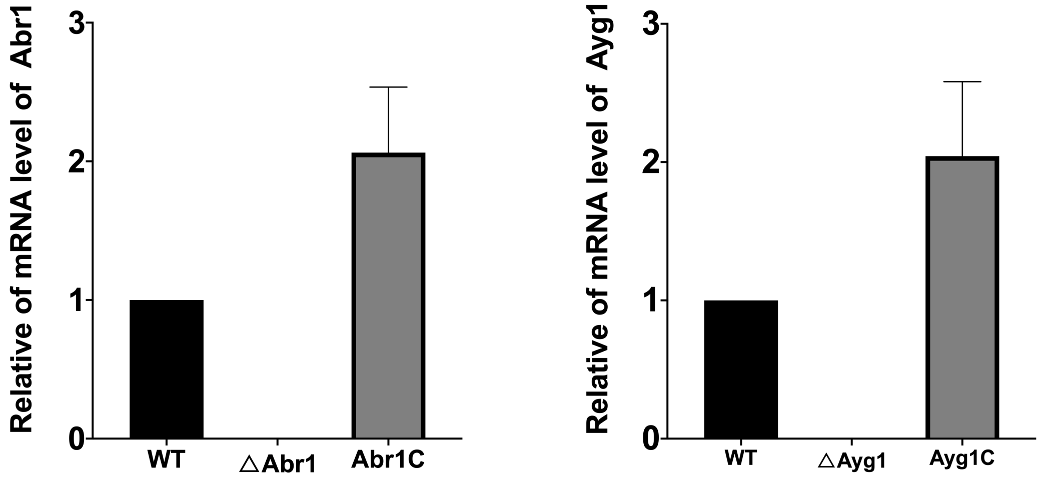

Supplement: FIG S3 [file mbio.03434-20-sf003.pdf]
