# Supplementary material for: Light Signaling Regulates Aspergillus niger Biofilm Formation by Affecting Melanin and Extracellular Polysaccharide Biosynthesis
Source: mBio. 2021 Feb 16;12(1):e03434-20. doi: 10.1128/mBio.03434-20 (PMC8545115; doi:10.1128/mBio.03434-20)
Supplement: TABLE S1 [file mbio.03434-20-st001.pdf]

Table S1 Sequence of the oligonucleotide primers used for gene knockout in this study.

Primer name	Primer sequence (5' to 3')	Source
Abr1-up-F	AAAGCAGCCGAGGCCTTATT	This work
Abr1-up-R	GGTGGAGGCGGCGGATTTTA	This work
	TGCCGCGGCTTCGAGTATAG	
Abr1-down-F	GCCCACTCCACATCTCCACT	This work
	CGATGGCTCCAAACTGACGTTTATCGG	
Abr1-down-R	CACTGCAACCAAACAGAGCC	This work
Hph- *Abr1*-F	CTATACTCGAAGCCGCGGCA	This work
	TAAAATCCGCCGCCTCCACC	
Hph- *Abr1*-R	CCGATAAACGTCAGTTTGGA	This work
	GCCATCGAGTGGAGATGTGGAGTGGGC	
Abr1-Check-F	GCATCCATATCGCCAAGGGT	This work
Abr1-Check-R	GGCGCTTCGATAATGATGGC	This work
Ayg1-up-F	CTGGGACACCGTCCATGTAG	This work
Ayg1-up-R	GGTGGAGGCGGCGGATTTTA	This work
	GGTGTTTGCTTGCTCGTGTCG	
Ayg1-down-F	GCCCACTCCACATCTCCACT	This work
	CGAAAATAAGGGTTATTTCCAACGCGTCG	
Ayg1-down-R	GCTCCACTGACGATATTTGCG	This work
Hph- *Ayg1*-F	CGACACGAGCAAGCAAACAC	This work
	CTAAAATCCGCCGCCTCCACC	
Hph- *Ayg1*-R	CGACGCGTTGGAAATAACCC	This work
	TTATTTTCGAGTGGAGATGTGGAGTGGGC	
Ayg1-Check-F	CATCTTCACACCGTCTACTC	This work
Ayg1-Check-R	TTCCGGTCAAGACATTGCGT	This work
