# Supplementary material for: Light Signaling Regulates Aspergillus niger Biofilm Formation by Affecting Melanin and Extracellular Polysaccharide Biosynthesis
Source: mBio. 2021 Feb 16;12(1):e03434-20. doi: 10.1128/mBio.03434-20 (PMC8545115; doi:10.1128/mBio.03434-20)
Supplement: TABLE S2 [file mbio.03434-20-st002.pdf]

Table S2 Sequence of the oligonucleotide primers used for plasmid construction in this study.

Primer name	Primer sequence (5' to 3')	Source
P*trpC*- *Abr1*-F	TAGAGGTAATCCTTCTTTCT AGATCGACAGAAGATGATATTGAAGGAGCAC	This work
P*trpC*- *Abr1*-R	GCGTTGTGAGGGTTCGGATC ATGGTTACTTCCTAATCGAAGCTTTGCT	This work
Abr1-F	ATGATCCGAACCCTCACAACGC	This work
Abr1-R	CAGTAACGTTAAGTGGATCC TAACTTGCAGGGAGGGAATACTTATCAG	This work
T*trpC*- *Abr1*-F	GATCCACTTAACGTTACTGAAATC	This work
T*trpC*- *Abr1*-R	CGACGGCCAGTGCCAAGCTT GCATTGCAGATGAGCTGTATCTGG	This work
P*trpC*- *Ayg1*-F	TAGAGGTAATCCTTCTTTCT	This work
P*trpC*- *Ayg1*-R	CCGAGGATCCAAGGAGCCAT GGTTACTTCCTAATCGAAGCTTTGCT	This work
Ayg1-F	ATGGCTCCTTGGATCCTCGG	This work
Ayg1-R	CAGTAACGTTAAGTGGATCC TAGTTCTTGAGAGGCTCCTGGG	This work
T*trpC*- *Ayg1*-F	GATCCACTTAACGTTACTGAAATC	This work
T*trpC*- *Ayg1*-R	CGACGGCCAGTGCCAAGCTT GCATTGCAGATGAGCTGTATCTGG	This work
