# Supplementary material for: Light Signaling Regulates Aspergillus niger Biofilm Formation by Affecting Melanin and Extracellular Polysaccharide Biosynthesis
Source: mBio. 2021 Feb 16;12(1):e03434-20. doi: 10.1128/mBio.03434-20 (PMC8545115; doi:10.1128/mBio.03434-20)
Supplement: TABLE S3 [file mbio.03434-20-st003.pdf]

Table S3 Genes and primers used for qRT-PCR.

Gene ID	Locus ID	Forward rimer sequence (5' to 3')	Reverse primer sequence (5' to 3')
Actin	ANI_1_106134	GGTCTGGAGAGCGGTGGTAT	GAAGAAGGAGCAAGGGCAGTG
ChsA	ANI_1_120124	ACTCTTTCTTGCCTCGTCGG	CACGCATAGTGCTGCCAATC
Chs1	ANI_1_1554084	GGCGGTTTTGGATATGCGAC	CGCATCACTGAGTTGAGGGT
ChsC1	ANI_1_1214104	ATCTCCGGCCGGCTATCTAT	GCATGACACCGTGAAGGGTA
ChsC2	ANI_1_684064	TGTTGACCCGATGTGTGGAG	GCCGACTCCAGAGGCTTATC
ChsD	ANI_1_1986074	TCAGATGTGCACGAGTGCTT	TGGGTAGCGTTTCCACAGTC
Gtb3	ANI_1_3050024	GCAAATACGCCGAAGACGAC	ACATGCGACGGGTGATCTTT
Agd3	ANI_1_3048024	CAGTACGACGTGTATCCGGG	CAGTCTTCAGGCCAGACGTT
Ega3	ANI_1_1582024	CGAGAATTGGCGTCCGGATA	TGCGAGGAGAGCTGATGTTG
Sph3	ANI_1_3046024	TGGAAGGGCACAAATTCCGA	TGGCTTCCAAAGCTCGCATA
Uge3	ANI_1_1578024	GATTTCCGGGATGTTGCTGC	TCGCTGTAGACCTCTTCCCA
Uge5	ANI_1_558124	AGAAGTGGAACGGTGCTCTG	GGAAGCAGGTTGTAGGGGAC
Ags1	ANI_1_1472184	GGTCTACCGTGAATGTGCCA	CAAAAGTGTTGCCCGAGGTG
Ags2	ANI_1_360084	AGGTGACCTCAAGCGTCAAG	ATCGACCAACGACCAACGAA
Fks1	ANI_1_188054	GCCCACTTTCACCGTCCTTA	GAGTACGGCTCATCCTCACG
Pksp/Alb1	ANI_1_726084	CCTTTCGTCTCAGCCCTGTT	TCAGCTTCGCATGCCTTGTA
Ayg1	ANI_1_740124	ACGGTGCCATCGAAGACTTT	AAAGCAGCCGAGGCCTTATT
Arp1	ANI_1_2396074	GCCGATGGGTATGATCGGAA	CCAAGTCTCGACGAAGTGGT
Arp2	ANI_1_224144	ATCGTCCGCAGTTTGAGTGA	CGGCTTCATGGTTGTCTTGC
Abr1	ANI_1_1496124	TCCACCGGAATTCATTGGCA	ACCGGTTTGGTTCATCGGAA
Abr2	ANI_1_1310034	TCCCGTGTCGTGGATTTCAG	GTTCACGCGGAGATTTGGTG
RodA	ANI_1_412064	CTGGTACCCTGTCCAACCTC	CCGATGATGGGGATCTGGAG
RlmA	ANI_1_1692024	AATACGTGCGTTGTCCACCT	CGTTAAAGTCTTCGGGGCCT
Hog1	ANI_1_826074	TTTCGTCACGGAACTCCTGG	GATCGCGATGAACAACACCG
MpkA	ANI_1_476164	TTCCAGATCCGCAGTTGCTT	AGCTTGACCTGACCATTCCG
